# Simultaneous two-photon activation and imaging of neural activity based on spectral–temporal modulation of supercontinuum light

**DOI:** 10.1117/1.NPh.7.4.045007

**Published:** 2020-11-03

**Authors:** Yuan-Zhi Liu, Carlos Renteria, Connor D. Courtney, Baher Ibrahim, Sixian You, Eric J. Chaney, Ronit Barkalifa, Rishyashring R. Iyer, Mantas Zurauskas, Haohua Tu, Daniel A. Llano, Catherine A. Christian-Hinman, Stephen A. Boppart

**Affiliations:** a University of Illinois at Urbana–Champaign, Beckman Institute for Advanced Science and Technology, Urbana, Illinois, United States; b University of Illinois at Urbana–Champaign, Department of Electrical and Computer Engineering, Urbana, Illinois, United States; c University of Illinois at Urbana–Champaign, Department of Bioengineering, Urbana, Illinois, United States; d University of Illinois at Urbana–Champaign, Neuroscience Program, Urbana, Illinois, United States; e University of Illinois at Urbana–Champaign, Computational Science and Engineering, Urbana, Illinois, United States; f University of Illinois at Urbana–Champaign, Department of Molecular and Integrative Physiology, Urbana, Illinois, United States; g University of Illinois at Urbana–Champaign, Carle Illinois College of Medicine, Urbana, Illinois, United States

**Keywords:** two-photon optogenetics, two-photon fluorescence microscopy, calcium imaging, neurons, supercontinuum, photonic crystal fiber

## Abstract

**Significance:**

Recent advances in nonlinear optics in neuroscience have focused on using two ultrafast lasers for activity imaging and optogenetic stimulation. Broadband femtosecond light sources can obviate the need for multiple lasers by spectral separation for chromatically targeted excitation.

**Aim:**

We present a photonic crystal fiber (PCF)-based supercontinuum source for spectrally resolved two-photon (2P) imaging and excitation of GCaMP6s and C1V1-mCherry, respectively.

**Approach:**

A PCF is pumped using a 20-MHz repetition rate femtosecond laser to generate a supercontinuum of light, which is spectrally separated, compressed, and recombined to image GCaMP6s (930 nm excitation) and stimulate the optogenetic protein, C1V1-mCherry (1060 nm excitation). Galvanometric spiral scanning is employed on a single-cell level for multiphoton excitation and high-speed resonant scanning is employed for imaging of calcium activity.

**Results:**

Continuous wave lasers were used to verify functionality of optogenetic activation followed by directed 2P excitation. Results from these experiments demonstrate the utility of a supercontinuum light source for simultaneous, single-cell excitation and calcium imaging.

**Conclusions:**

A PCF-based supercontinuum light source was employed for simultaneous imaging and excitation of calcium dynamics in brain tissue. Pumped PCFs can serve as powerful light sources for imaging and activation of neural activity, and overcome the limited spectra and space associated with multilaser approaches.

## 1 Introduction

The use of multiple optical tools for imaging and excitation of neurons and neural tissue has grown dramatically in recent years. Researchers have successfully engineered two-photon (2P) systems for bidirectional excitation and measurement of neural activity with sophisticated optical designs.^1^^–^^7^ Typically, two spectrally distinct laser sources are required to reliably and independently excite fluorophores and optogenetic probes with minimal crosstalk. With a large palette of optogenetic proteins, calcium indicators, voltage indicators, and other custom-tailored indicators to monitor and induce neural activity^8^^–^^13^ comes the added need of multiple laser sources for bidirectional probing of neural tissue. This becomes even more striking with the ubiquitous use of ultrafast laser sources, which are needed to induce 2P absorption events, and thus elicit responses from individual neurons and brain regions due to the reduced out-of-focus excitation.^3^^,^^14^^–^^19^ Indeed, the reduced out-of-focus excitation, which can be exploited with high spatiotemporal resolution for single-cell interrogation of neural circuits, provides an added level of control for brain research that is otherwise not possible with continuous wave (CW) illumination. This allows for the single-cell precision needed for directed, optogenetic excitation of neural circuits. These sources, however, add to the increased cost, space, and complexity of these systems, and consequently the technical expertise necessary to operate and maintain these systems. For these systems to become more widely used in the neuroscience community, more versatile systems are required. Furthermore, the broad spectrum of fluorophores of interest to neuroscientists requires spectral tuning to realize the versatility that comes with this diversity.

Advances in supercontinuum generation provide such promise. Multiphoton processes typically require ultrafast light sources to achieve the high-peak power needed to generate responses with longer-wavelength sources. Ultrafast sources have become increasingly adopted for optical imaging^20^^–^^24^ to track inherent metabolic and structural properties of tissue for applications in oncology,^23^^–^^28^ biophysics,^29^^–^^33^ and neuroimaging.^4^^,^^12^^,^^18^^,^^34^^–^^37^ Notably, the broad spectrum of activity indicators and optogenetic proteins in neural studies, although convenient and versatile, typically require multiple laser sources. These setups are highly specific to the spectral constraints imposed by the light source. Multiple setups or dramatic changes, and thus extensive system realignment, to existing setups are required, especially for multiphoton setups. This becomes even more difficult should multiple fluorophores or optogenetic proteins with distinct spectral bands be used for experiments. These limitations can be overcome by using a coherent supercontinuum optical source and spectral–temporal pulse shaping.

Coherent supercontinuum light sources can be generated by carefully designed photonic crystal fibers (PCFs)^38^^–^^42^ and multispectral amplitude and phase shaping of light can be implemented for selective excitation of photosensitive molecules. Notably, pulse shaping using a 4f pulse shaper has provided the ability to reliably and selectively excite fluorophores of interest, while suppressing undesired signals.^20^ Although fiber-based lasers are increasing in popularity for bioimaging,^21^^,^^25^^,^^27^^,^^38^^,^^43^^,^^44^ the advantages of supercontinuum generation and pulse shaping are otherwise not widely implemented for microscopy. Beyond spectral selectivity, amplitude, and phase shaping of fiber-based supercontinuum facilitate imaging by providing high-power, short pulses^45^^–^^48^ that improve the efficiency of the nonlinear optical processes we elicit.^45^ These advances and advantages of supercontinuum sources, however, have not been widely recognized in neuroscience, with most groups adopting the use of multiple femtosecond lasers for their investigations.^1^^,^^2^^,^^6^^,^^7^

In this work, we developed a multiband 2P excitation system based on supercontinuum generation for simultaneous 2P calcium imaging and 2P optogenetic excitation of neural tissue. A femtosecond fiber laser was used to pump a PCF for supercontinuum generation, followed by pulse-shaping of dual spectral bands to generate near transform-limited pulses, as done previously in our group.^21^ A spatial-light-modulator-based pulse shaper was used for spectral–temporal femtosecond pulse shaping in this work, which provides a user-friendly method for simultaneous multispectral band pulse compression that otherwise would require multiple separate pulse compressors with delicate alignment of optical dispersive elements for the specific spectral bands. This pulse shaper solely requires the user to select the spectrum of interest and to operate the corresponding pulse compression with a computer.^49^ These two individually compressed spectral bands were guided into two optical paths, one for the excitation of the calcium indicator GCaMP6s in brain tissue,^50^ and the other for the optogenetic protein C1V1-mCherry.^51^ The hippocampi of transgenic GCaMP6s mice were bilaterally injected with C1V1-mCherry using a commercial AAV virus for optogenetic expression of the excitatory opsin, C1V1-mCherry. The brain slices were harvested after at least 3 weeks postinjection and used for experiments. Our results demonstrate positive expression of these spectrally distinct fluorophores using the supercontinuum source and actuation of single-cell activity using optogenetics. Continuous-wave lasers were used to verify the widespread excitation of activity in the brain and verify the localization of excitation seen using our ultrafast PCF source. Spiral-scanning was implemented to elicit a response from individual cell bodies.^7^ This work shows the utility of supercontinuum generation in neuroscience as an alternative to multilaser systems.

## 2 Methods

All animal procedures were performed in accordance with the relevant guidelines and regulations under an IACUC protocol approved by the University of Illinois at Urbana–Champaign.

### 2.1 Viral Injection and Brain Slice Preparations

A C1V1-mCherry transducing viral vector [AAV-DJ-CaMKIIa-C1V1-(E122T/E162T)-TS-P2A-mCherry, Stanford Neuroscience Gene Vector and Virus Core] was injected into the dentate gyrus of hippocampi in transgenic Thy1-GCaMP6s mice (Stock # 025776, Jackson Labs) of both sexes, 1 to 3 months old (P30 to P90). Animals were euthanized up to 3 months postinjection for experiments. Animals were anesthetized with 2% to 3% oxygen-vaporized isoflurane (Clipper Distributing Company) and placed in a stereotaxic apparatus (Kopf Instruments). Carprofen (5  mg/kg, Zoetis) was administered subcutaneously prior to surgery. The viral solution was loaded into a 10-μL Nanofil syringe with a 33G needle driven by an injection pump controller (Micro4, World Precision Instruments). A 1-μL volume of the viral solution at a titer of $∼10^{12}\;\text{genomes}/\mathrm{mL}$ was injected into the hippocampal dentate gyrus region (1.8 mm posterior to bregma, ±1.3  mm lateral from bregma, and 1.4 ventral to the cranial surface) at 0.12  μL/min. The syringe was left stationary 3 to 5 min postinjection to promote diffusion of the viral vector throughout the injection site. The wound was closed with silk sutures (Perma-hand, Ethicon). Finally, 2.5% lidocaine + 2.5% prilocaine cream (Hi-Tech Pharmacal) and Neosporin (Johnson & Johnson) were applied topically to the injection site to promote healing and reduce inflammation.

For slice preparation, animals were anesthetized by 2% to 3% oxygen-vaporized isoflurane, followed by decapitation. The mouse brain was then isolated and quickly transferred to chilled cutting solution (254 mM sucrose, 11 mM glucose, 2.5 mM KCl, 1.25 mM ${\mathrm{NaH}}_{2}{\mathrm{PO}}_{4}$, 10 mM ${\mathrm{MgSO}}_{4}$, 0.5 mM ${\mathrm{CaCl}}_{2}$, and 26 mM ${\mathrm{NaHCO}}_{3}$). A vibratome (VT1000S, Leica Biosystems) was then used to cut 300-μm-thick slices. Regions near the injection site were saved for experimentation, whereas all other brain slices were discarded. Sliced tissue was incubated in artificial cerebrospinal fluid (126 mM NaCl, 2.5 mM KCl, 10 mM glucose, 1.25 mM ${\mathrm{NaH}}_{2}{\mathrm{PO}}_{4}$, 1 mM ${\mathrm{MgSO}}_{4}$, 2 mM ${\mathrm{CaCl}}_{2}$, and 26 mM ${\mathrm{NaHCO}}_{3}$ at ∼298  mOsm) at 32°C for 60 min, then transferred to room temperature for at least 15 min prior to imaging. Slices were continuously perfused with 95% oxygen and 5% ${\mathrm{CO}}_{2}$ at all times.

### 2.2 Calcium Imaging and Single-Cell Photostimulation Experiments

**Fig. 1:**
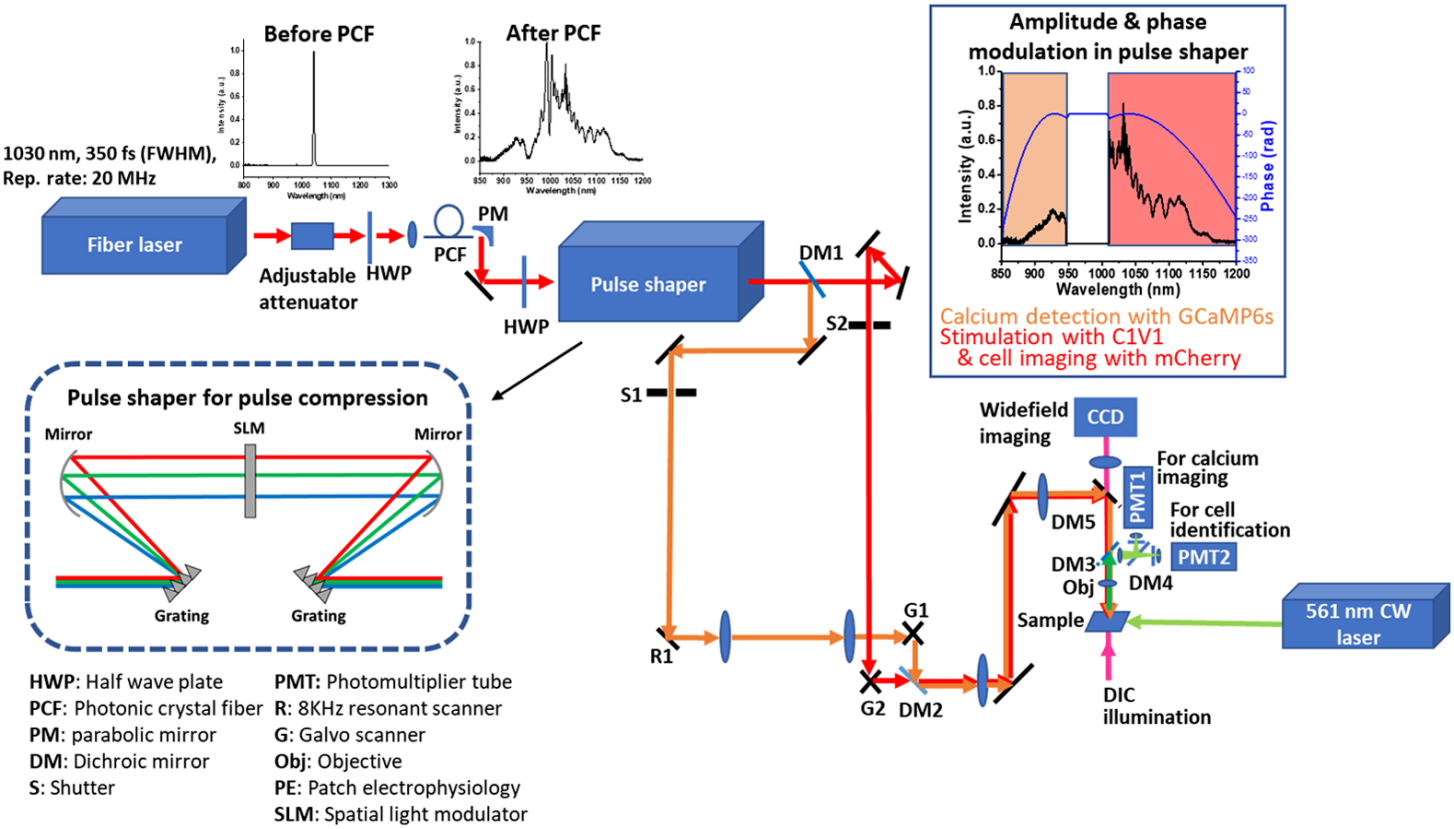
Optical system used for experiments. A 1030-nm, 350-fs, and 20-MHz repetition rate laser is used to pump a PCF for supercontinuum generation. The light is directed to a pulse shaper which compresses the pulses at two bands to maximize 2P efficiency and improve image quality. After the pulse shaper, the light is split with a dichroic mirror to separate paths. The GCaMP6s path is driven by a resonant scanner paired to galvanometers for high-speed imaging. The C1V1-mCherry path is directed to a pair of galvanometers to scan for imaging and for spiral scanning of target neurons. The two paths are recombined at the sample for experiments. Separately, a 561-nm CW laser was directed by a multimode optical fiber to the sample for widefield optogenetic activation.

The experimental setup is illustrated in Fig. 1. A large mode area PCF (PM-LMA-15, NKT Photonics) was pumped by a 1030-nm femtosecond fiber laser (Satsuma, Amplitude Systemes) to generate the highly polarized coherent fiber supercontinuum.^21^ Although the pump laser can output an average power of 10 W, we only applied 3.5 W for pumping, which was more than sufficient for our experiments. The repetition rate was also tuned to 20 MHz in these studies. The broadband coherent supercontinuum was directed to a 640 pixel, 4-f pulse shaper (MIIPS Box 640, Biophotonics Solutions Inc.) for further spectral–temporal modulation. The broadband spectrum was separated into two bands (890 to 950 nm and 1010 to 1160 nm) inside the pulse shaper by a knife-edge-based amplitude modulation, which minimized the excitation crosstalk between GCaMP6s and C1V1-mCherry, as shown in the inset of Fig. 1. With the programmable capability of the spatial light modulator inside the pulse shaper, optimized band-specific phases were simultaneously applied to these two spectral bands to achieve individually near transform-limited pulse compressions (890 to 950 nm, ∼51  fs and 1010 to 1140 nm, ∼35  fs). This approach of temporal modulation is more convenient and versatile compared to the common pulse compressor methods, e.g., prism/grating pairs, which require two separate sets with careful alignment, along with the need to be realigned for different spectral band applications. The relatively broad bandwidth of each spectral band, along with the low pulse repetition rate of 20 MHz, was advantageous for generating higher peak power compared to standard 80 MHz pulses, as reported in our previous work.^21^ This becomes more explicit with the formal relation between peak power $P_{\text{peak}}$, average laser power $P_{\mathrm{avg}}$, pulse duration $t_{p}$, and laser repetition rate $f_{p}$ defined as $P_{\text{peak}}=\frac{P_{\mathrm{avg}}}{t_{p}f_{p}}$. Because higher peak power can reduce the average laser power requirement for 2P excitation and minimize phototoxicity and photobleaching, the low repetition rate strategy has also recently begun to be utilized within the neuroscience community.^6^^,^^7^^,^^17^

The beam after the pulse shaper was then split into two paths by a 980-nm long-pass filter (FF980-Di01, Semrock) (DM1) for excitation of GCaMP and C1V1-mCherry, which were recombined (DM2) after the scanners and directed (DM5) to a modified Olympus BX51 microscope. Calcium imaging was performed at video rate using an 8-kHz resonant scanner (SC-30, Electro-Optical Products) for one transverse axis with a standard galvanometric-scanned mirror (6215H, Cambridge Tech) for the second transverse axis. For C1V1-mCherry imaging and excitation, the beam was scanned using a pair of galvanometric-mounted mirrors. Fluorescence from calcium imaging was detected during point-scanning using an analog PMT (H7422A-40, Hamamatsu) then amplified using a PMT Transimpedance amplifier (TIA60, ThorLabs). For C1V1-mCherry imaging, the fluorescence was detected using a photon counting PMT (H7421-40, Hamamatsu). All images covered a 300  μm×300  μm field-of-view. A movable dichroic mirror (DM3) was placed to separate the near-infrared excitation beam from the fluorescence for PMT detection (735 nm high pass, Semrock), with an additional dichroic placed to separate the GCaMP and C1V1-mCherry fluorescence (593 nm high pass, Semrock) in front of the PMT. The DM3 mirror can be slid out for wide field fluorescence and DIC imaging with a CCD camera (QImaging Retiga Electro). Filters were implemented for detection of GCaMP6s and C1V1-mCherry fluorescence at 510±42  nm and 641±37.5  nm, respectively (FF01-510/84-25 and F-F02-641/75-25, Semrock). The sample was illuminated with ∼18  mW of power for excitation and imaging of C1V1-mCherry. About 18 mW of power was used for imaging GCaMP6s in brain tissue. Experiments were also conducted with a CW laser source operating at a 561-nm wavelength (Sapphire, Coherent). A partially under-filled objective (XLPlan N 25× 1.05 NA, Olympus) was used for all experiments. Previous studies have demonstrated that a lower effective numerical aperture, rather counter-intuitively, improves 2P optogenetic activation in brain tissue due to the increased number of excited opsins.^52^^,^^53^

For optogenetic experiments, the brain tissue was first examined by widefield GCaMP imaging, followed by C1V1-mCherry imaging, to verify the presence of GCaMP6s and determine regions with dense C1V1-mCherry expression. Once a region was established, the optogenetic stimulation protocol (both CW and 2P) was used to evoke responses from cells near transduced areas. A spiral scanning approach was implemented for 2P stimulation, with controlled exposure times, as reported in Table 1. For CW stimulation, widefield illumination across the region of interest was employed, targeting all cells in the field-of-view. Stimulation protocols were initiated after a wait time of at least 1 min after identifying C1V1-mCherry positive regions to promote recovery of the proteins to their resting, ground state. Similarly, cells illuminated with either CW or 2P illumination were given adequate time (>1  min) to recover prior to subsequent stimulation. The response was then visualized in real time and the data were recorded for postprocessing. All data were processed offline using custom-written MATLAB software. All hardware for imaging and stimulation protocols were controlled using a custom-written LabVIEW program.

**Table 1:** Stimulation protocols for Fig. 4.

Protocol	Exposure (ms)	Cells targeted	# Stimulations	# Epochs	Burst?
CW: P1	500	All	1	5	No
CW: P2	500	All	1	5	No
CW: P3	500	All	1	5	No
2P: P1	30	i and ii	20	1	Yes
2P: P2	50	v	1	5	No
2P: P3	200	ii	1	5	No

### 2.3 Image Denoising

To reduce the presence of noise inherent in fluorescent microscopy systems, image denoising was implemented prior to image analysis. In particular, the Noise2Void denoising technique^54^ was implemented to remove sources of noise from the captured calcium imaging sequences. Calcium transients were visible without the algorithm, but the image quality was dramatically improved, which allowed for ready identification of sources of these dynamic changes. The algorithm was run in PyCharm. Representative calcium imaging datasets demonstrating the efficacy and reliability of Noise2Void are shown in Fig. S1 in the Supplementary Materials. A representative video demonstrating the effectiveness of denoising is also included in Video S1 [URL: https://doi.org/10.1117/1.NPh.7.4.045007.1]. Additionally, the signal-to-noise ratio is quantified before and after denoising in Fig. S2 in the Supplementary Materials, quantified using the equation, $\mathrm{SNR}=10\;\log_{10}(\frac{\mu_{\mathrm{sig}}}{\mu_{\mathrm{bckg}}})$, where $\mu_{\mathrm{sig}}$ and $\mu_{\mathrm{bckg}}$ represent the mean intensity from an image representative of signal and background, respectively.

### 2.4 Data Analysis

All analyses were performed offline using custom MATLAB software, using a previously devised algorithm.^55^ Calcium transients were isolated from individual cells by selecting a point on the soma and averaging around a five-pixel crosshair. Raw images were saved in binary format. Videos were processed with a mean projection along time using ImageJ to generate the final images, unless otherwise noted. Stimulation times were validated by measuring the image power throughout the time series.

## 3 Results

### 3.1 Imaging and Transduction Validation

**Fig. 2:**
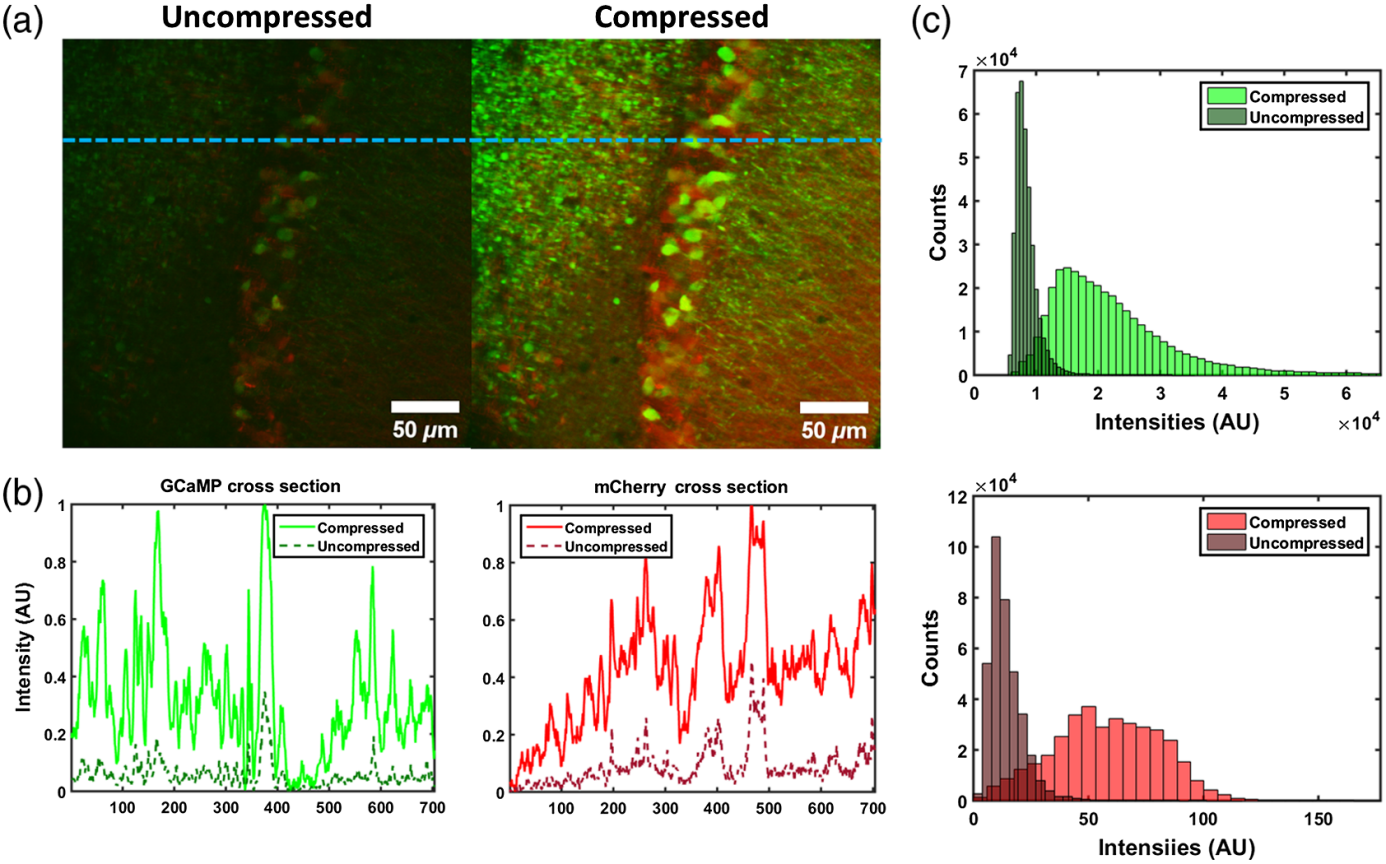
Image pulse compression characterization. (a) Images of hippocampal brain slices imaged with the optical setup show a dramatic increase in image quality after pulse compression. (b) A cross section of the image (green and red) is also plotted with and without pulse compression for both the intended GCaMP6s and C1V1-mCherry channels, respectively. The cross sections were normalized to the peak intensity for the compressed images. (c) The histograms also quantify the considerable increase for both the GCaMP (top) and mCherry (bottom) images, before and after pulse compression. Notably, the distribution of intensities from the samples are much broader and illustrate a clear increase in signal intensity overall. Scale bars represent 50  μm; n=1 slice, 1 mouse.

Initial experiments were designed to ensure that the imaging parameters were optimal for both beam paths from the supercontinuum source. Brain slices from GCaMP6s mice injected with C1V1-mCherry were imaged with and without pulse compression to demonstrate image quality optimization. Results highlighted in Fig. 2 show images of these cells and consequently the validation of the expected emission for the desired spectral bands. In particular, Fig. 2(a) shows fluorescence from hippocampal tissue both before and after pulse compression, where the green channel is GCaMP6s and the red channel is mCherry. The results verify both that adequate detection of sufficient image quality can be obtained with the system and that pulse compression does indeed result in improving the signal strength via enhanced 2P absorption efficiency. The line plots in Fig. 2(b), which correspond to the region highlighted by the blue dashed line in Fig. 2(a), show a cross section of the image that further exemplifies the increased signal obtained by pulse compression. Histograms of the pixel intensities of these images also illustrate the dramatic increase in signal after pulse compression. Figure 2(c) clearly illustrates a larger distribution of higher intensity pixels after pulse compression for both GCaMP6s and C1V1-mCherry. The presence of visible structures in the sample in Fig. 2(a), along with the line plots in Fig. 2(b) and histograms in Fig. 2(c), together highlight the efficacy of the system for the desired wavelengths, and the improved image quality following pulse compression optimization.

**Fig. 3:**
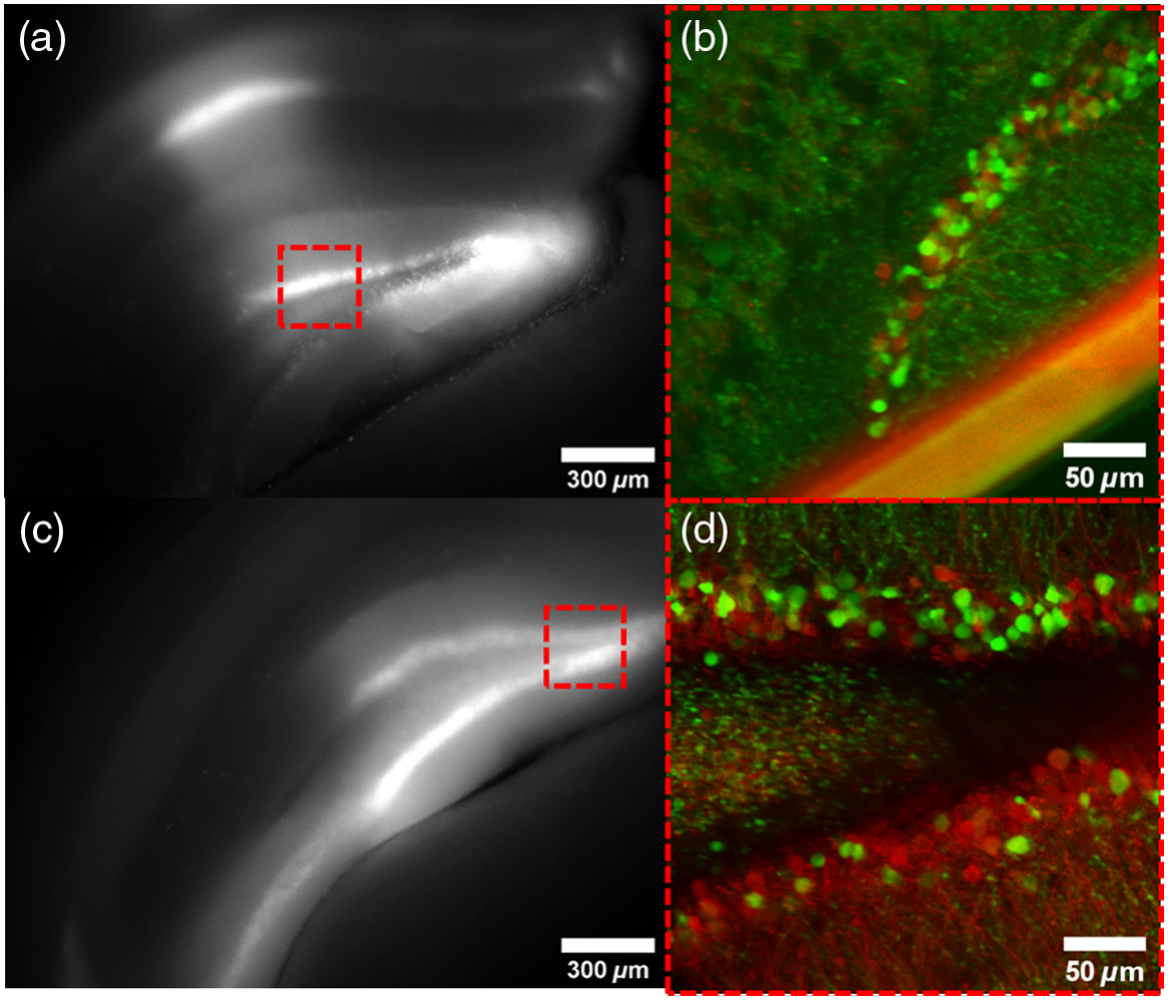
Brain tissue expressing GCaMP and C1V1-mCherry. (a), (c) Widefield fluorescence imaging of two separate slice preparations shows very bright fluorescence in distinct regions of the hippocampus. (b), (d) Magnified 2P images corresponding to the areas in the red dashed boxes in the widefield images on the left illustrate positively transduced cells throughout the mouse brain at different brain sites. A wire harp was used to keep the tissue stationary, and one wire strand is shown in (b) as a large diagonal bar across the hippocampus. (a), (c) 4× scale bars represent 300  μm. (b), (d) 20× scale bars represent a distance of 50  μm; n=2 slices, 2 mice.

Similarly, brain slices harvested from GCaMP6s-positive mice were imaged under the microscope to verify image quality and successful transduction of the targeted sites. Figures 3(a) and 3(c) show widefield fluorescence images of C1V1-mCherry from the harvested brain slices using a 4× objective (Olympus). Here we see positive expression of C1V1-mCherry in neurons, which verifies the virus reached the intended delivery site. Further multiphoton imaging with the optical setup in Figs. 3(b) and 3(d) shows expression of GCaMP-positive neurons, in addition to structures of the hippocampus from C1V1-mCherry. An additional slice preparation in Fig. 3(c), coupled with the 2P imaging results from Fig. 3(d), demonstrates the reliability of the system for producing high-quality images for the fluorophores of interest. Collectively, these results demonstrate the capabilities of the system to visualize the intended fluorescent structures at targeted injection sites.

### 3.2 Simultaneous Imaging and Stimulation

**Fig. 4:**
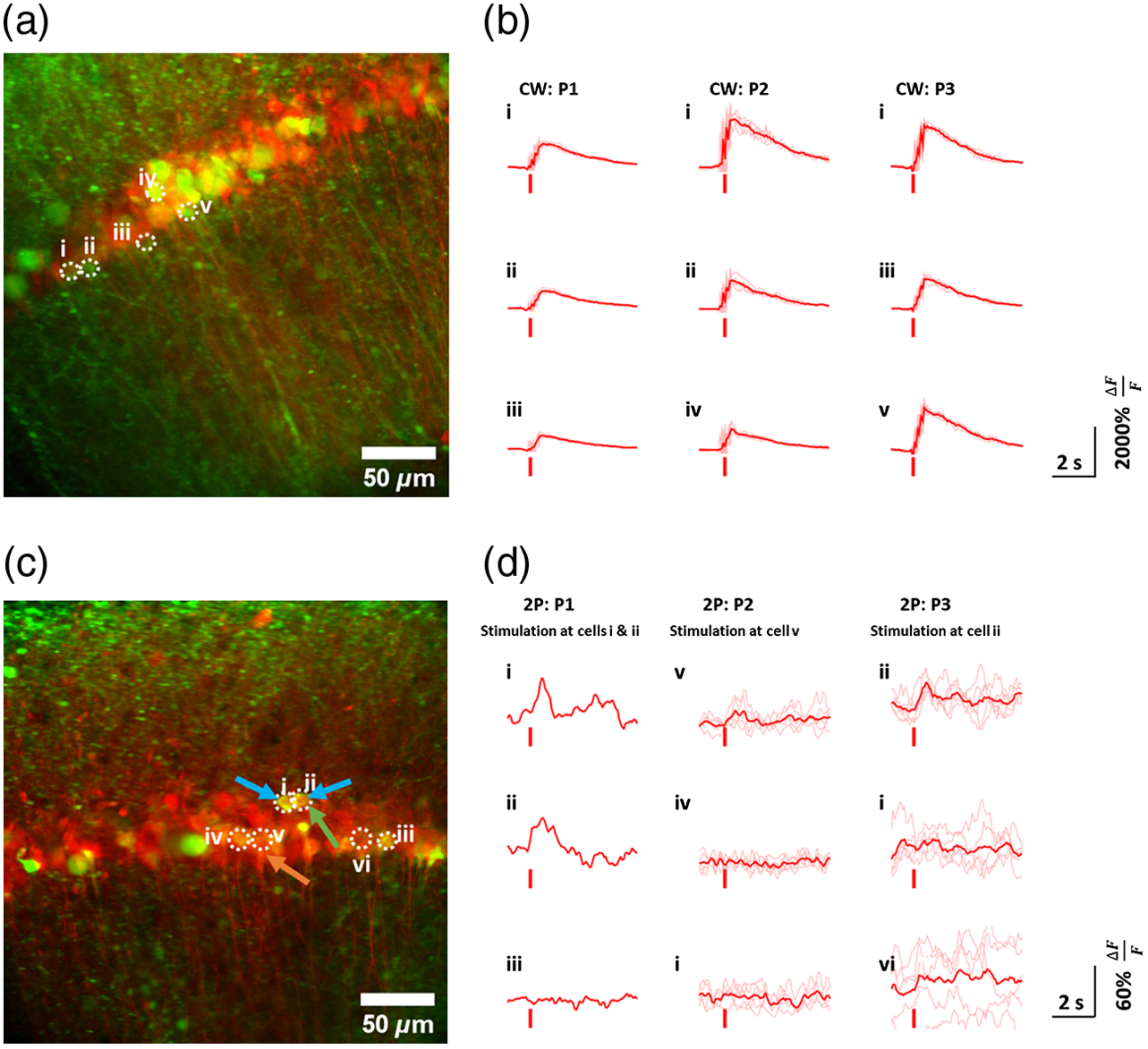
Photoactivation of GCaMP using (a), (b) single-photon excitation of C1V1-mCherry and (c), (d) from multiphoton excitation. The red tick marks in (b) and (d) represent the time of the optical stimulus. Three separate stimulation protocols for CW (CW: P1 to P3) and 2P stimulation (2P: P1 to P3) were run for each sample. For CW excitation, there was widefield illumination (b), whereas for 2P protocols (d), a single area was targeted. In (c), the blue arrows highlight the cells excited for protocol 2P: P1, the orange arrow indicates the targeted cell for protocol 2P: P2, and the green arrow indicates the cell targeted in 2P: P3. Cells were labeled between (i) and (vi), and their calcium signals are plotted for each dataset (b), (d). Red traces represent the mean signal from the calcium traces plotted in pink. Data from protocol P1 under 2P stimulation (d) had a single, burst stimulation epoch, and hence, no individual trials. CW light was incident for 500 ms. Multiphoton sources were incident on the sample for 30 ms for protocol P1, 50 ms for protocol P2, and 200 ms for protocol P3. Image scale bars represent 50  μm; n=5 cells under CW stimulation; and n=6 cells under multiphoton stimulation. Both CW and multiphoton protocols were performed on the same brain slice. Calcium plot scale bars represent 60% (2P) or 2000% (single photon) $\frac{\Delta F}{F}$ on the y axis, and 2 s on the x axis.

Finally, verification that this system could simultaneously perform 2P calcium imaging and 2P excitation of C1V1-mCherry was performed. Expression for mCherry is shown in Figs. 3(a) and 3(c), and both GCaMP6s and C1V1-mCherry are shown in the multiphoton images in the remainder of Fig. 3, as well as in Figs. 4(a) and 4(c). Sites visibly present with GCaMP6s, C1V1-mCherry, and co-expression of the two can thus be easily identified. Expression of GCaMP6s can clearly be seen throughout the tissue and targeted delivery of C1V1-mCherry expresses well throughout the targeted region of the hippocampus. These validate, again, that targeted delivery of the virus and the genetic expression was successful. Next, actuation of the opsin was validated by excitation with a CW laser source. As previous literature has shown that the efficiency for absorption in opsins is significantly higher for CW excitation than 2P,^52^ this was performed as an initial validation that the opsin was functional. Stimulation protocols for both CW and 2P illumination are summarized in Table 1 for ease of reference. Figure 4(b) shows the calcium response of wide-field excitation of the transduced sample with a 561-nm CW laser ($∼2\;\mathrm{mW}/{\mathrm{mm}}^{2}$), corresponding to the locations in Fig. 4(a). Immediately, a dramatic increase in signal from GCaMP6s was evoked and captured from dozens of cells. Plots from three representative cells are shown in Fig. 4(b), with the average response (red) across multiple stimulation epochs ($n_{\text{epochs}}=5$) superimposed on individual trials (pink). The experiment was repeated three times [Fig. 4(b), 1P: P1 to P3] to demonstrate the repeatability of these responses. These data verify the presence of each probe of interest, and the capability of both eliciting and detecting neural responses. Furthermore, they illustrate the capability of monitoring dynamic calcium transients, optogenetically evoked or otherwise.

Finally, single-cell excitation and imaging using the supercontinuum source was tested. Initial reference images of GCaMP6s and C1V1-mCherry were acquired and used to guide localization of the stimulation beam. Thereafter, a cell of interest was selected, and the stimulation protocol was initiated. Two-photon-excited imaging of GCaMP6s was performed at a 13.12-Hz framerate, whereas 2P stimulation pulses with 30, 50, and 200 ms exposure were applied to cells i/ii, v, and ii, respectively. The results are shown in Figs. 4(c) and 4(d), where protocol 2P: P1 corresponds to the 30-ms stimulus applied to cells i and ii, 2P: P2 the 50-ms stimulus at cell v, and 2P: P3 to the 200-ms stimulus at cell ii. In our studies, protocols were defined by the cells targeted, the scanning geometry, and the stimulation time, as detailed in Sec. 2 and Table 1. Protocol 2P: P1 had only a single-stimulation epoch, compared to five for all other protocols. Previous literature has demonstrated that repeated 2P optical stimuli in a given timeframe result in more reliable calcium responses,^7^ and this tested for this particular protocol. For ease of reference, all cells stimulated under different protocols at the same site are identified in Fig. 4(c), with the blue arrows specifying cells stimulated under protocol 2P: P1, the orange arrow specifying the identified cell for protocol 2P: P2, and the green arrow the cell targeted under protocol 2P: P3. Spiral scanning was implemented for excitation of the entire cell to elicit sufficient 2P responses. Notably, the calcium transients for each protocol resulted from a single or pair of activated cell(s) near the stimulation site, rather than from several as with CW excitation. This suggests the beam was localized to a designated spot as intended for each protocol, and consequently, resulted in cells within a confined region being excited. This is especially clear for protocols 2P: P2 and 2P: P3, where cells v and ii, respectively, were targeted and showed a clear response, coupled with consistent increases in intensity for each stimulation epoch as well. In contrast, cells iv and i for protocol 2P: P2 and cells i and vi in protocol 2P: P3 did not respond. When a targeted region encompassing two neighboring cells was activated, as in Fig. 4(d) (protocol 2P: P1), there was an increase in fluorescence from both cells i and ii at the time of stimulation, with an absence thereof for cell iii. This provides further support that the targeted regions alone resulted in a calcium response. Unlike the widefield CW illumination, 2P protocols targeted different single cells. These targeted regions, again, show cells at the site of interest that responded to the optical stimulus. More specifically, cells i and ii in protocol 2P: P1, cell v in protocol 2P: P2, and cell ii in protocol 2P: P3 all showed a clear and consistent response to 2P activation. In each case, the targeted cell showed, on average, an increase in fluorescence.

Notably, when cell ii was stimulated individually, in contrast to simultaneously stimulating both cells i and ii in protocol 2P: P1, only cell ii showed a clear response. Figure 4(d) along protocol 2P: P1 targeted both cells for stimulation, whereas protocol 2P: P3 targeted only cell ii. Also the calcium signals evoked with CW excitation are considerably higher than those for 2P activation, which is consistent with reduced amplitudes from patch-clamp recordings reported from other groups.^52^^,^^56^ These results provide evidence that in addition to imaging, supercontinuum generation is useful for neural applications and optogenetics, reducing the need for complex multilaser systems, associated space occupation, and flexibility with regards to multispectral excitation.

## 4 Discussion

Presented here is a novel supercontinuum light source and system for simultaneous, multispectral excitation, detection, and imaging of neural activity. Although recent work has demonstrated supercontinuum sources for nonlinear imaging, none has shown the ability to use these sources for simultaneous neural excitation and imaging. Rather, multispectral imaging studies have used a definitive band of the supercontinuum that is optimal for excitation of multiple fluorophores and sufficient for detection of multiharmonic signals.^21^ This work demonstrates the utility of supercontinuum sources beyond this, using spectrally distinct beam paths from the same source to elicit excitation of spectrally distinct molecules. This is critical in simultaneous imaging and stimulation experiments to avoid undesired excitations, such as exciting opsins with the imaging beams.^1^^,^^7^^,^^57^ Supercontinuum sources promote flexibility for sufficient spectral separation to minimize crosstalk. The benefit of multiphoton excitation for reduced out-of-focus excitation, coupled to the flexibility of spectral separation, makes them ideal for these experiments. Moreover, the reduced cost of using a single ultrafast laser for these types of experiments reduces financial barriers associated with the multilaser approaches. The PCF used in this work is commercially available and costs $95 for the length of fiber used. The dichroic mirror used to split the supercontinuum light is about $750. Compared to purchasing two lasers, this is a much more economical approach. However, it should be noted that the pulse shaper used in this work is a high-end model and comparable to the cost of a separate laser. It does, however, provide the versatility for precise spatiotemporal control of ultrafast pulses and consequently a high degree of control over experimental variables. For these types of experiments, however, a high-end pulse shaper is not required. Near transform-limited pulses can easily be generated using chirped mirrors or prism pairs and are widely used in multiphoton microscopy for pulse compression. Use of these pulse-compression methods with a supercontinuum source, in theory, still manage to maximize beam quality while circumventing the need for a second laser source, which may still require the implementation of these compression techniques regardless. Thus the added multispectral capabilities from a single supercontinuum source can be easily and inexpensively generated.

Coherent supercontinuum sources provide additional benefits beyond spectral selectivity with regards to optogenetics. The literature has shown previously that compared to CW optogenetic excitation, 2P excitation has shown poorer efficiency for eliciting neural responses. The first experiments demonstrating 2P excitation of single cells theoretically and experimentally showed that 2P excitation, although capable of eliciting action potentials, resulted in weaker peak currents and voltages than monochromatic sources.^52^ This is attributable to several factors, including the excitation volume, the 2P absorption cross section of channelrhodopsin, exposure time, power, and various other factors. These have since been overcome through spiral scanning and spatial–temporal wavefront shaping to cover entire cell somas and temporal shaping to modify the focal volume along the optical axis.^58^ Each of these comes with their own detriments, including reduced spectral bandwidth due to dispersion in the case of temporal focusing,^4^ and the dependence on reliable scanning geometries and decreased speed for scanning approaches.^7^^,^^19^ Still, the benefit of reliable single-cell activation and the simultaneous activation of ensembles of cells with single-cell, high temporal resolution that can be realized with 2P illumination, far outweighs the detriment imposed by the lower peak currents. Furthermore, use of longer-wavelength light sources promotes an increased penetration depth that is advantageous for deep tissue and *in vivo* imaging. Invasive surgical procedures are necessary for CW illumination to target a directed brain region and are still limited by increased out-of-focus excitation. Multiphoton sources, in contrast, overcome both limitations. Recent work has demonstrated that temporal phase shaping of the excitation pulse, rather than removal of spectral information, is not only sufficient for eliciting excitations, but also modifies the resulting response from the whole-cell.^33^ This suggests that with carefully tailored optical pulses, this 2P excitation efficiency can be overcome or otherwise altered, and even be used to elicit different responses on a single and multicell level.

This coherent control principle, coupled with the multispectral and compressible capabilities of supercontinuum sources, could in theory be used to optimize the excitation efficiency of a broad array of opsins. This principle has also been demonstrated in fluorescence imaging,^20^ but much less investigated in opsins and similar photosensitive proteins. Further research will investigate the capabilities and implications of this combined approach, especially in the context of interneural communication. Still, supercontinuum sources are limited in that they require regular maintenance and skilled personnel to accompany their use. Fiber quality needs to be optimized, maintained, and replaced when damaged. Although maintenance is straightforward, training to ensure PCF output quality is required for new personnel. As this technology is furthered, efforts toward facile implementation that overcomes the need for skilled personnel will be pursued. Additionally, output power after a PCF is dependent on the coupling efficiency of the fiber and power for each spectrum on nonlinear conversion during supercontinuum generation. This, again, requires trained personnel to optimize, and a system design that must be based on the output spectrum that can be provided by the fiber. This design consideration can be limiting if insufficient power is available for a desired spectral band.

Multicell, multispectral excitation could, in theory, be used with multiple opsins to excite, inhibit, and track neural dynamics. Metabolic information can be tracked simultaneously with optogenetic excitation to track the energy demands that different excitation parameters place on neural tissue, just as it has in other settings^23^^,^^59^^–^^62^ and can be readily implemented using PCFs. For multicell excitation experiments, a sufficiently large amount of power is needed to provide adequate power spectral density for optogenetic activation.^7^^,^^18^ In our study, after splitting the beam, we find ∼90  mW can be obtained for the 920-nm band and ∼500  mW for the 1060-nm band. Only a small fraction of this power budget is used in the experiments here. Higher pump power also provides higher output power of supercontinuum, if necessary. Given the high image quality from the GCaMP6s channel, there is more than enough power in the imaging band to facilitate real-time imaging of calcium transients. Conveniently, the laser source used in this study has a tunable repetition rate, which further facilitates investigations of the laser parameters used in microscopy studies. With trends toward lower repetition rate lasers, power budget limitations can be more easily overcome to promote adequate excitation of multiple cells in a sample by the inherent increase in 2P absorption. By experimenting with the optical parameters, including illumination volume, scanning trajectory, repetition rate, pulse width, and power among other variables, optimal parameters can be determined to optimize 2P optogenetic activation. By leveraging these, more sophisticated neural circuit experiments can be realized.

## 5 Conclusions and Outlook

We present a single-source approach for imaging and optogenetic control of neural activity using supercontinuum generation. Stereotaxic injections in the hippocampi of transgenic GCaMP6s mice with C1V1-mCherry were performed to test the utility of this source for all-optical neural interrogation. The presence of C1V1-mCherry was verified in the designated injection site with 2P imaging and was used to guide 2P neural activation. CW excitation was performed to verify that neural tissues were expressive and responsive. Two-photon excitation was then performed while imaging GCaMP6s transients, and localized cellular responses were elicited. These results demonstrate the utility of supercontinuum and PCFs for neuroscience studies, which would benefit the neuroscience community by providing a flexible and less expensive alternative to traditional multilaser multiphoton setups.

## 6 Appendix

Video S1 compares representative calcium imaging data before (left) and after (right) denoising.

## 7 Data, Code, and Material Availability

The code used to generate the results in this manuscript is available from the corresponding author upon reasonable request and through collaborative investigations.
