# Supplementary material for: Simultaneous two-photon activation and imaging of neural activity based on spectral–temporal modulation of supercontinuum light
Source: Neurophotonics. 2020 Nov 3;7(4):045007. doi: 10.1117/1.NPh.7.4.045007 (PMC7607614; doi:10.1117/1.NPh.7.4.045007)
Supplement: Supplementary file 1 [file NPh_007_045007_SD001.pdf]

Supplementary Files:

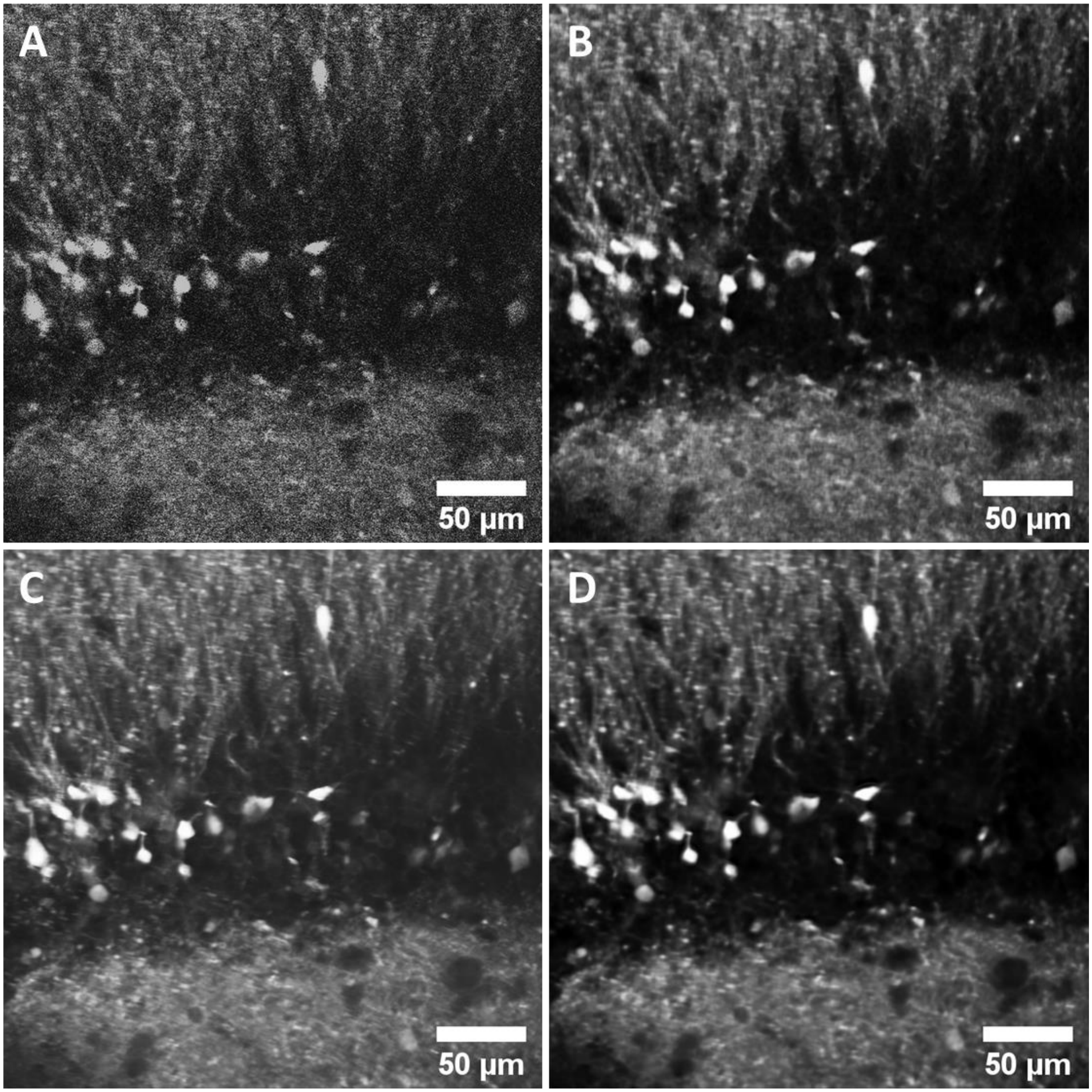

Supplementary Figure 1: Comparison of raw and denoised two-photon images. A representative frame from one of the experiments from the raw data (A) and denoised data (B). Projections of each video are also highlighted for the raw (C) and denoised datasets (D).

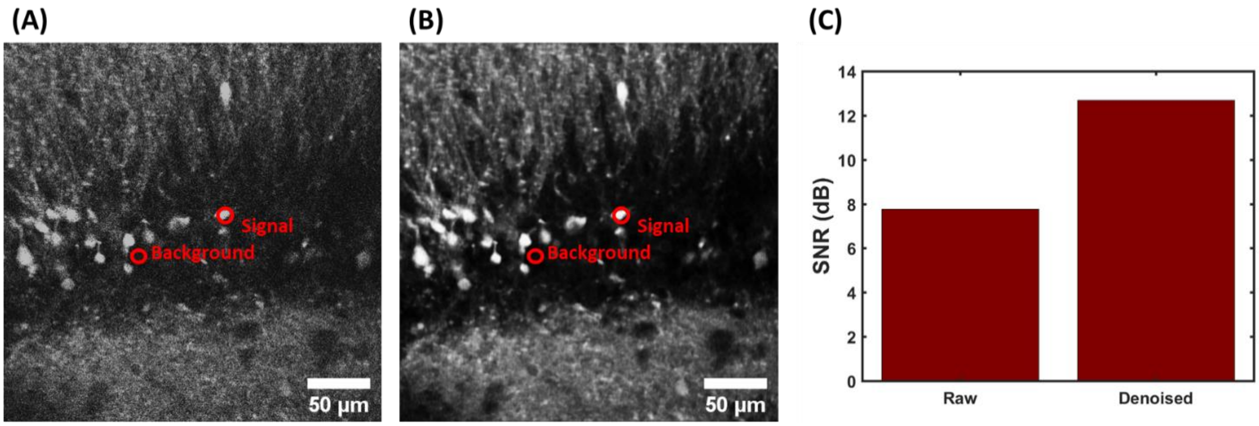

Supplementary Figure 2: Further comparison of raw and denoised two-photon images. The original images (A) and denoised image (B) from one of the datasets was used to quantify the increase in signal-to-noise ratio (SNR) after denoising (C). The region chosen to represent the signal and background used to quantify SNR are labelled in (A) and (B). A nearly 5 decibel increase SNR from 7.78 to 12.69 after denoising is quantified in this dataset.
